# Comparison of Keverprazan-based versus esomeprazole-based dual therapy for initial treatment of *Helicobacter pylori* infection: a prospective, multicenter, randomized controlled trial

**DOI:** 10.1080/07853890.2026.2706993

**Published:** 2026-08-09

**Authors:** Zhaoli Ma, Xi Gou, Hongjuan Ma, Hong Lu, Min Liu, Hao Yuan, Qiang Li, Jun Wang, Huizhen Yang, Yuling Wang, Zhaofeng Chen, Xiaochuang Shu, Yue Zhao, Chen Li, Wenjin Li, Ya Zheng, Rui Ji, Qian Ren

**Affiliations:** ^a^ The First School of Clinical Medicine, Lanzhou University, Lanzhou, Gansu Province, China; ^b^ Department of Gastroenterology, The First Hospital of Lanzhou University, Lanzhou, Gansu Province, China; ^c^ Gansu Province Clinical Research Center for Digestive Diseases, Lanzhou University, Lanzhou, Gansu Province, China; ^d^ Department of Gastroenterology, Dingxi People’s Hospital, Dingxi, Gansu Province, China; ^e^ Department of Gastroenterology, Tanchang People’s Hospital, Longnan, Gansu Province, China

**Keywords:** *Helicobacter pylori*, dual therapy, potassium-competitive acid blocker, Keverprazan, esomeprazole, eradication rate

## Abstract

**Background:**

Keverprazan offers a new perspective for *Helicobacter pylori* eradication. This study compared 14-day keverprazan-amoxicillin therapy with esomeprazole-amoxicillin therapy to explore a superior treatment strategy.

**Methods:**

This was a prospective, open-label, multicenter, randomized controlled trial in adult patients with treatment-naive *H. pylori* infection. Participants were randomly assigned to receive either 14-day of KA therapy (Keverprazan 20 mg b.i.d plus amoxicillin 1 g t.i.d) or 14-day of EA therapy (Esomeprazole 40 mg b.i.d plus amoxicillin 1 g t.i.d). The primary outcome was the *H. pylori* eradication rate. Secondary outcomes were the incidence of adverse events and patient adherence.

**Results:**

A total of 264 patients were enrolled in the study. In the intention-to-treat (ITT) analysis, the eradication rates for the 14-day KA group and the 14-day EA group were 87.9% and 80.3%, respectively (*p* = 0.092); in the modified intention-to-treat (mITT) analysis, the eradication rates were 92.1% and 86.2%, respectively (*p* = 0.135); and in the per-protocol (PP) analysis, the eradication rates were 93.5% and 88.3%, respectively (*p* = 0.155). Non-inferiority was confirmed between the two groups (all *p* < 0.001). Adverse events and patient adherence were similar between the two groups.

**Conclusion:**

For treatment-naive *H. pylori* infection, the 14-day KA therapy is non-inferior to EA therapy. Given its good tolerability, pharmacogenomic independence, and potent acid suppression, KA is a rational first-line alternative to EA in the Chinese population.

## 1. Introduction

*Helicobacter pylori* (*H. pylori*) is a Gram-negative, microaerophilic bacterium. As a major pathogen causing chronic gastritis, peptic ulcers, and gastric cancer, its infection poses a serious threat to human health [1]. Data indicate that the global prevalence of *H. pylori* infection has exceeded 50% [1]. Multiple large-scale, long-term follow-up studies have confirmed that eradicating *H. pylori* not only significantly reduces the incidence of gastric cancer but also demonstrates a significant cumulative time-dependent effect. That is, the longer the duration after eradication, the greater the benefit [2–4]. Based on the above evidence, the Kyoto Global Consensus Report on *H. pylori* Gastritis explicitly states that, unless there are competing factors, all *H. pylori*-positive individuals should receive treatment [5].

Currently, clinical treatment for *H. pylori* infection primarily involves two regimens. The first-line option is bismuth-containing quadruple therapy (BQT), which combines a proton pump inhibitor (PPI), bismuth, and two antibiotics [6–9]. The alternative regimen is clarithromycin-based triple therapy, consisting of a PPI combined with clarithromycin and amoxicillin (or metronidazole) [10]. Although these regimens initially achieved eradication rates exceeding 90%, their success rates have shown a gradual decline due to factors such as increased antibiotic resistance and insufficient acid suppression [11,12]. Furthermore, the non-standardized selection of antibiotics in clinical practice is a key factor leading to eradication failure and the development of drug resistance, thereby increasing the rate of secondary resistance and ultimately contributing to the growing prevalence or ‘refractory *H. pylor* infection’ in clinical settings [13].

In recent decades, extensive and in-depth research on *H. pylori* eradication therapies has been conducted both domestically and internationally. The core objective of these studies has consistently focused on identifying an ideal treatment regimen that balances high eradication rates, high patient compliance, and a low incidence of adverse reactions. Against this backdrop, high-dose dual therapy (HDDT) has gradually emerged as a research focal point, serving as a highly promising alternative regimen. HDDT primarily involves the combination of a PPI with high-dose amoxicillin. Previous randomized controlled trials (RCTs) have directly compared the clinical efficacy of bismuth-containing quadruple therapy with high-dose PPI-amoxicillin dual therapy. The results showed that the eradication rates and adverse event incidence were similar between the two groups, suggesting that HDDT possesses potential comparable to that of traditional therapies[14]. In addition to RCTs, comprehensive data from multiple meta-analyses have also strongly confirmed this conclusion [15,16]. In light of this, multiple international guidelines currently recommend this HDDT regimen as a first-line clinical treatment option [7,17]. However, the efficacy of HDDT has not been consistent across all studies. For instance, a study by Graham et al. conducted in the United States involving a 14-day high-dose, high-frequency PPI dual therapy reported an eradication rate of only 72%, falling far short of the expected ideal outcome [18]. In PPI-based regimens, even with high-dose and high-frequency administration strategies, the acid suppression effect may still be limited by the pharmacokinetic properties of PPI, failing to reach the optimal acid suppression level required for *H. pylori* eradication. Therefore, we believe that acid suppression therapy plays a crucial role in eradication regimens. Potent and sustained acid suppression enhances antibiotic efficacy and stability by increasing intragastric pH, ensuring effective drug concentrations [19]. With continuous advancements in drug development, novel potassium-competitive acid blockers (P-CAB) have emerged. Compared with traditional PPI, P-CAB offer significant advantages, including faster onset, more prolonged action, and superior acid suppression, enabling a more rapid and stable increase in intragastric pH. The advent of P-CABs overcomes the acid suppression bottleneck of traditional therapies, promising further improvements in *H. pylori* eradication rates.

Keverprazan (KPZ) is the first P-CAB independently developed in China, having received approval in February 2023 [20]. In terms of pharmacokinetics, compared with traditional PPIs, KPZ exhibits more rapid, potent, and sustained acid suppression characteristics [21]. Existing evidence suggests that this pharmacological advantage may translate into improved *H. pylori* eradication efficacy, particularly in the context of antibiotic resistance. Clinical studies have also confirmed that KPZ-containing quadruple therapy demonstrates satisfactory efficacy and safety in first-line treatment [22]. However, to date, there is a lack of studies directly comparing the clinical efficacy of KPZ-based dual therapy versus PPI-based dual therapy.

To evaluate the efficacy of KPZ-based dual therapy in Chinese patients, we conducted a prospective study comparing 14-day Keverprazan-amoxicillin (KA) dual therapy with esomeprazole-amoxicillin (EA) therapy in Chinese patients, aiming to provide an effective, safe, and convenient strategy for *H. pylori* eradication.

## 2. Methods

### 2.1. Study design

This study is a prospective, multicenter, randomized controlled, open-label, non-inferiority clinical trial. The study was conducted concurrently across three medical centers in Gansu Province: The First Hospital of Lanzhou University, People’s Hospital of Dingxi, and People’s Hospital of Tanchang County in Longnan. This study strictly adhered to the principles of the Declaration of Helsinki and the guidelines of the CONSORT statement for randomized controlled trials. The study protocol was approved by the Ethics Committee of The First Hospital of Lanzhou University (Approval No. 2024-08-07). Furthermore, this trial has been registered at ClinicalTrials.gov (Registration No. NCT06851468). All participants provided signed informed consent for participation in this study.

This study consecutively enrolled patients with initial *H. pylori* infection who visited the aforementioned three hospitals between March 2025 and September 2025. The specific inclusion criteria were as follows: 1) aged 18 to 65 years, regardless of gender; 2) diagnosis of *H. pylori* infection confirmed by a ^13^C or ^14^C urea breath test; 3) no prior history of *H. pylori* eradication therapy; 4) signed informed consent for participation in this study. The exclusion criteria were as follows: 1) presence of severe systemic diseases, or dysfunction of vital organs such as the heart, lung, brain, liver, or kidney, or other serious illnesses including malignant tumors; 2) presence of active peptic ulcer complicated by bleeding, perforation, obstruction, or malignant transformation, or a current or past history of Zollinger-Ellison syndrome; 3) history of allergy to penicillin or any of the drugs used in this study regimen; 4) use of acid suppressants, including PPI, P-CAB, or H_2_ receptor antagonists (H_2_-RAs) within 2 weeks before enrollment, or use of antibiotics within 4 weeks before enrollment; 5) pregnant or lactating women; 6) any other condition that, in the opinion of the investigator, rendered the patient unsuitable for participation in this study.

### 2.2. Randomization and intervention

After signing the informed consent form, patients who met the inclusion criteria were randomly assigned to the KA or EA treatment group in a 1:1 ratio using a computer-generated random number table. The KA group received KPZ 20 mg twice daily combined with amoxicillin 1000 mg three times daily for a 14-day course. KPZ was taken 30 min after breakfast and dinner, while amoxicillin was taken 30 min after meals. The EA group received Esomeprazole (ESO) 40 mg twice daily combined with amoxicillin 1000 mg three times daily for a 14-day course. ESO was taken 30 min before breakfast and dinner, while amoxicillin was taken 30 min after meals.

Before the initiation of treatment, the patients’ baseline data were recorded in detail, and the patients were fully informed of the treatment regimen details and the importance of taking medication regularly. During the treatment period, the researchers conducted follow-ups *via* telephone, WeChat, or other methods on day 7 and day 14 to assess the occurrence of adverse reactions and medication adherence. For mild to moderate adverse events, the patients were instructed to continue taking the medication and were given symptomatic treatment. If severe adverse reactions occurred, the patients were advised to discontinue the medication immediately and visit the outpatient clinic. Additionally, the patients were instructed to strictly quit smoking and abstain from alcohol during the medication period. At least 4 weeks after the completion of treatment, a ^13^C or ^14^C urea breath test was repeated to evaluate the *H. pylori* eradication status.

### 2.3. Outcomes

The primary outcome measure of this study was the *H. pylori* eradication rate at least 4 weeks after the end of treatment, defined as the proportion of patients with a negative result on the ^13^C or ^14^C urea breath test. The study calculated and compared eradication rates using intention-to-treat (ITT) analysis, modified intention-to-treat (mITT) analysis, and per-protocol (PP) analysis, and calculated 95% confidence intervals (CI). The specific definitions were as follows: The ITT analysis set included all enrolled patients, among whom those lost to follow-up or who did not complete the UBT re-examination were classified as treatment failures; the mITT analysis set included patients who received at least one dose of medication and completed the urea breath test follow-up; the PP analysis set excluded patients who violated the protocol, such as those with less than 80% medication consumption.

The secondary outcome measures of this study included the incidence of adverse events, treatment adherence, and factors influencing eradication rates. During the follow-up period, researchers recorded any adverse events that occurred during the treatment process *via* telephone or WeChat. Specific symptoms included abdominal pain, abdominal bloating, diarrhea, constipation, nausea/vomiting, dry mouth, bitter taste, headache, dizziness, pruritus, rash, dyspepsia, etc., and they were graded and reported according to the impact of the adverse events on daily life. Adverse reactions were categorized as ‘mild (the adverse reaction is temporary, tolerable, and does not affect daily life)’, ‘moderate (the adverse reaction can cause psychological or physical discomfort and partially affects daily life)’, and ‘severe (the discomfort is serious, leading to the inability to conduct daily life normally)’. The frequency was assessed by counting the number of patients who experienced various types of adverse events; if the same patient experienced multiple adverse events, each was counted separately. Adherence was measured by calculating the ratio of the actual medication consumption during the treatment period to the total prescribed dosage for 14 days (calculated by counting remaining tablets); an adherence rate of ≥80% was determined as good adherence, while <80% was determined as poor adherence.

### 2.4. Sample size estimation

Sample size estimation was performed using PASS 15.0.5 software. This study aimed to explore the non-inferiority of the KPZ-containing regimen for *H. pylori* eradication in treatment-naive patients. Based on previous studies, we estimated the eradication rates for the KA and EA groups to be 90.2% and 88.5%, respectively [23,24]. With a non-inferiority margin of 10%, *α* = 0.025 (one side), 1−*β* = 0.80, the appropriate sample size was calculated as 110 per group. Considering a dropout rate of 20%, the final sample size was determined to be 132 cases per group.

### 2.5. Statistical analysis

Statistical analysis was performed using SPSS 26.0 and SAS 9.4 software. Normally distributed continuous data were expressed as mean ± standard deviation $\left(\bar{x}\pm s\right)$, and comparisons between groups were performed using Student’s *t*-test. non-normally distributed continuous data were expressed as median and interquartile range [M(*P*_25_, *P*_75_)], and comparisons between groups were performed using the Mann–Whitney *U* test. Categorical data were expressed as numbers or percentages (%), and comparisons between groups were performed using the *χ*^2^ test or Fisher’s exact probability test. The Clopper–Pearson method was used to calculate the primary efficacy endpoints and their 95% CIs for each group, and the Miettinen & Nurminen method was used to calculate the treatment difference between the two groups and the corresponding 95% CI. Non-inferiority testing was conducted using SAS software *via* a one-sided *Z*-test and one-sided 95% CI; if the lower limit of the 95% confidence interval for the difference in eradication rates was > −10% and *p* < 0.025, the result was considered to meet the non-inferiority criteria. Except for the non-inferiority test, *p* < 0.05 was considered statistically significant.

## 3. Results

### 3.1. Baseline characteristics

**Figure 1.**
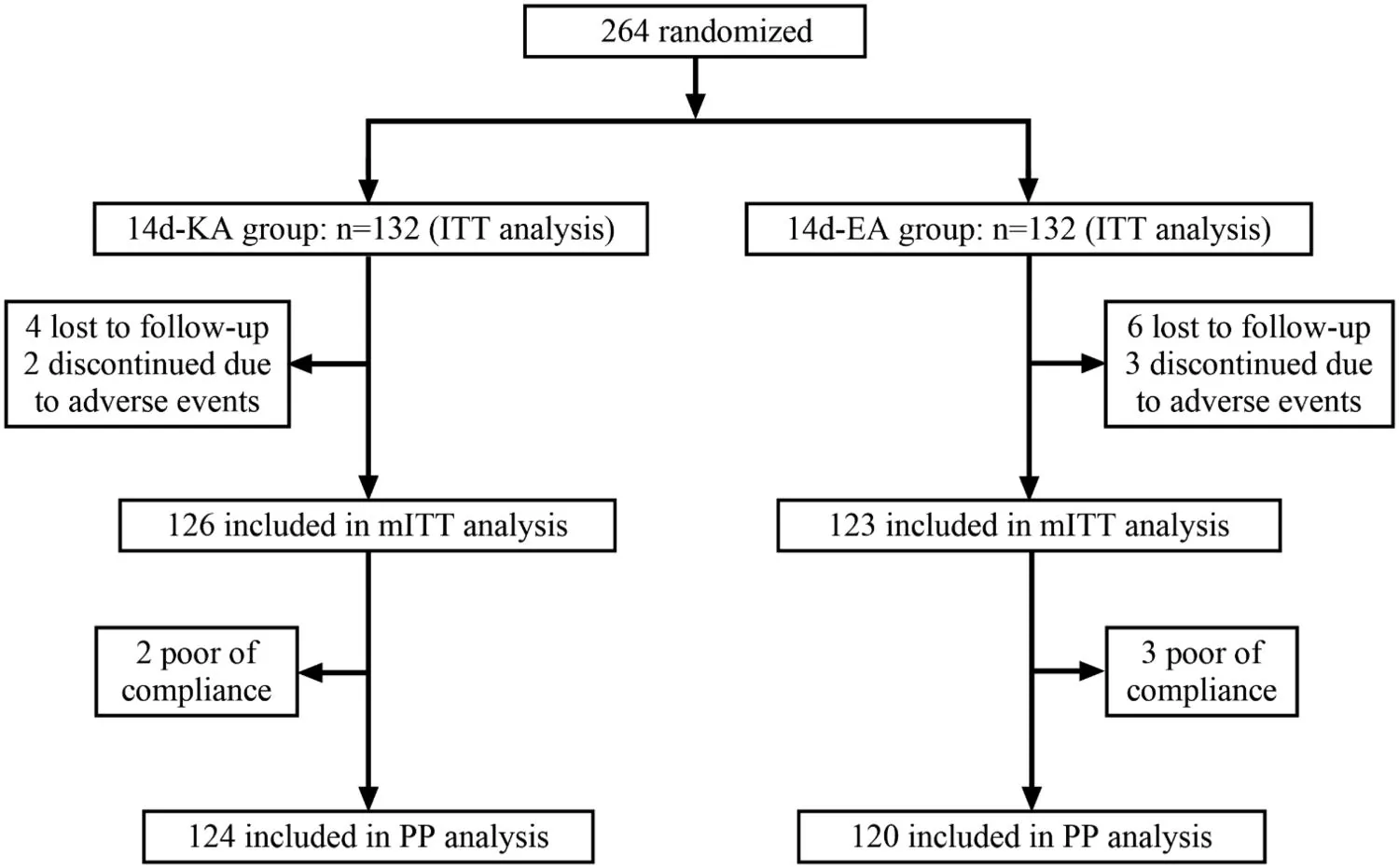
Flow diagram of this study. 14d-KA group, 14d keverprazan and amoxicillin dual therapy; 14d-EA group, 14d esomeprazole and amoxicillin dual therapy; ITT: intention-to-treat; mITT: modified intention-to-treat; PP: per-protocol.

As shown in Figure 1, a total of 264 *H. pylori*-positive subjects were enrolled in the study and randomly assigned in a 1:1 ratio to the 14-day KA group and the 14-day EA group, with 132 cases in each group. In the KA group, 4 patients were lost to follow-up, 2 patients withdrew from the study after discontinuing medication due to adverse reactions, and 2 patients took less than 80% of the prescribed medication. In the EA group, 6 patients were lost to follow-up, 3 patients withdrew from the study after discontinuing medication due to adverse reactions, and 3 patients took less than 80% of the prescribed medication. The baseline demographic and clinical characteristics of the two groups (including gender, age, body mass index [BMI], body surface area [BSA], marital status, smoking, alcohol consumption, dining habits, etc.) are summarized in Table 1. There were no statistically significant differences in baseline characteristics between the two groups (all *p* > 0.05).

**Table 1.** Baseline demographics and clinical data of patients.

	14d-KA group	14d-EA group	
Baseline factors	(*N* = 132)	(*N* = 132)	*p* value
Gender (male: female)	63:69	56:76	0.387
Age (years)	41.97 ± 12.46	41.36 ± 12.94	0.670
BMI (kg/m^2^)	22.72 ± 3.24	22.59 ± 3.57	0.241
BSA (m^2^)	1.68 ± 0.17	1.66 ± 0.18	0.719
Marital status (married: unmarried)	111:21	107:25	0.516
Smoking (often: seldom: has quit smoking: no smoking)	25:8:4:95	16:4:7:105	0.201
Drinking (often: seldom: has quit drinking: no drinking)	9:22:6:95	11:19:4:98	0.834
Style of dining (gather dining: individual dining)	106:26	101:31	0.455
Time of *H. pylori* infection diagnosis (in a month: <1 year: ≥1 year)	106:9:17	90:19:23	0.056
Gastroscopy within the past 6 months (gastritis: ulcer: not performed)	45:11:76	36:7:89	0.233
History of gastrointestinal disease (gastritis: ulcer: none)	36:8:88	33:8:91	0.914
Family history of *H. pylori* infection (yes: no: unknown)	31:45:56	34:44:54	0.911
Family history of gastrointestinal disease (gastritis: ulcer: gastric carcinoma: none)	14:6:2:110	12:5:3:112	0.868
History of antibiotics use in 2 years (yes: no)	75:57	73:59	0.804
Penicillin	61:71	62:70	0.902
Cephalosporins	51:81	40:92	0.154
Metronidazole	5:127	2:130	0.250
Clarithromycin	2:130	3:129	0.652
Furazolidone	1:131	2:130	0.561
Levofloxacin	14:118	12:120	0.171

### 3.2. *H. pylori* eradication rates

**Figure 2.**
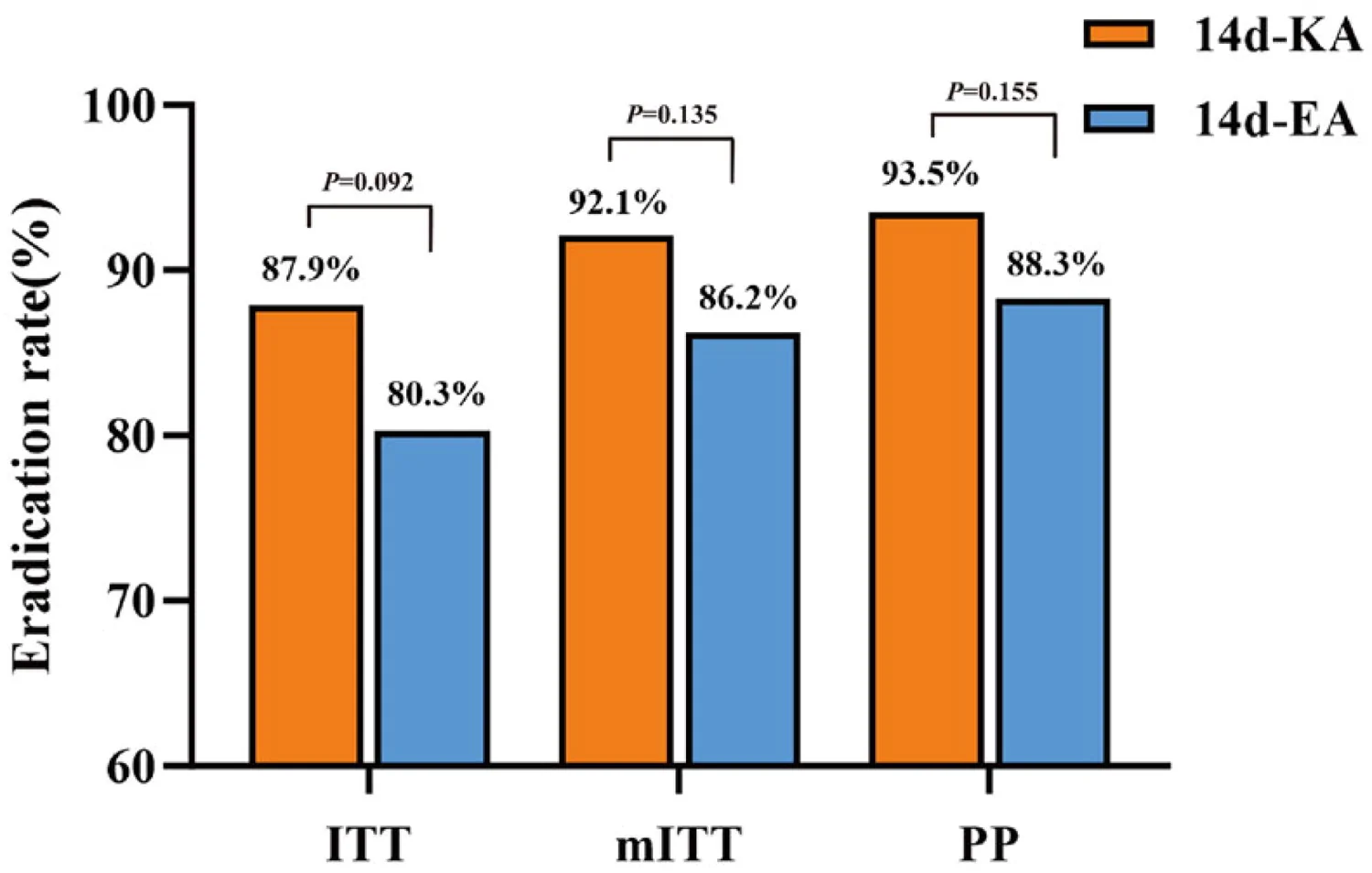
*H. pylori* eradication rates. 14d-KA, keverprazan and amoxicillin dual therapy; 14d-EA, esomeprazole and amoxicillin dual therapy; ITT: intention-to-treat; mITT: modified intention-to-treat; PP: per-protocol.

**Figure 3.**
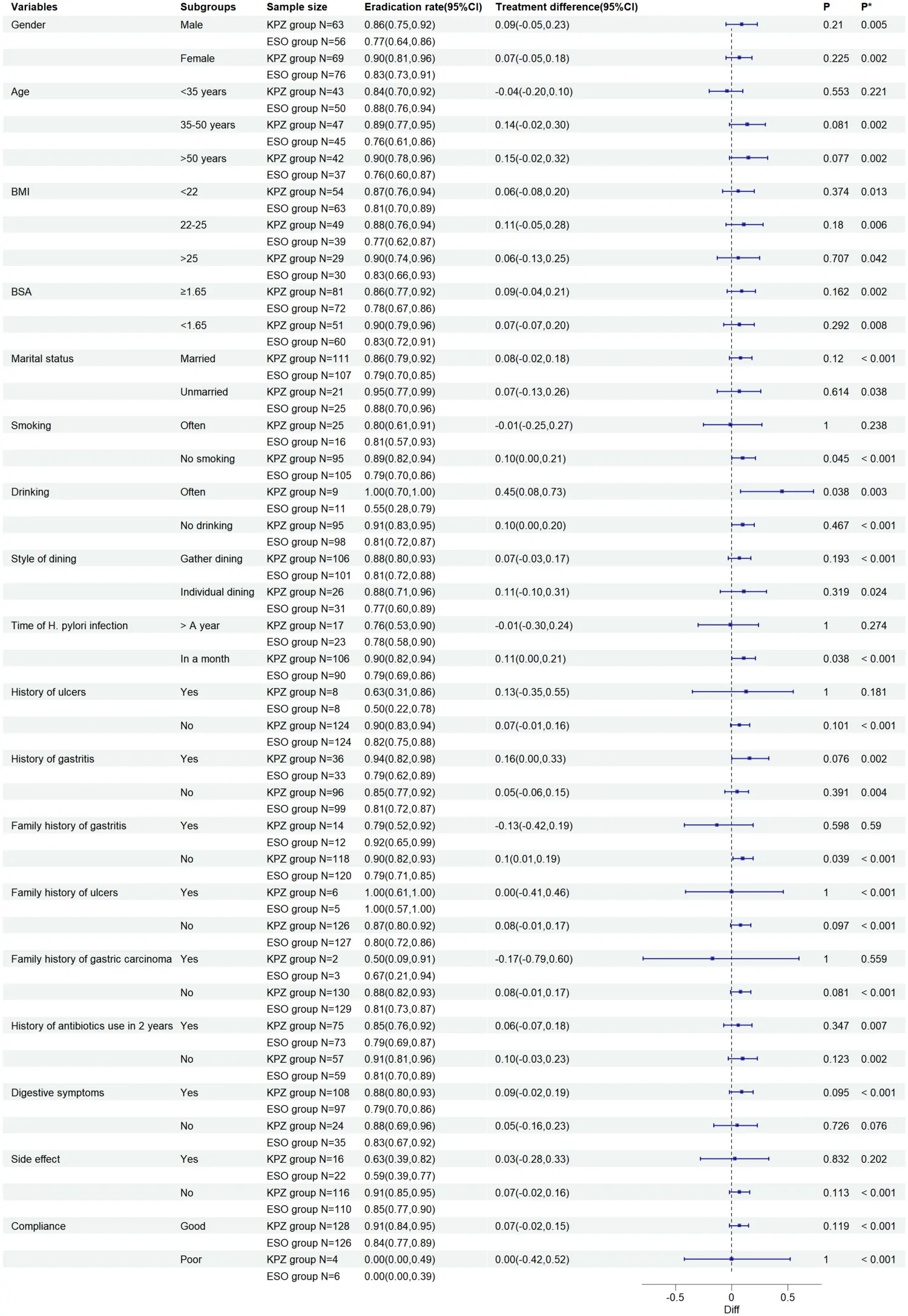
*H. pylori* eradication rates in subgroups (intention-to-treat population). CI: confidence interval; KPZ: keverprazan; ESO: esomeprazole.

The *H. pylori* eradication rates are detailed in Table 2 and Figure 2. In the ITT analysis, the eradication rates for the 14-day KA group and the 14-day EA group were 87.9% (116/132) and 80.3% (106/132), respectively; in the mITT analysis, the eradication rates were 92.1% (116/126) and 86.2% (106/123), respectively; and in the PP analysis, the eradication rates were 93.5% (116/124) and 88.3% (106/120), respectively. Statistical analysis confirmed that non-inferiority was established between the two groups (*p* < 0.001 for all analyses). In addition, the results of the subgroup comparative analysis between the two groups (Figure 3) showed that although in some subgroups the non-inferiority test did not reach the preset significance level, possibly due to the reduced sample size, but the overall trend indicated that compared with the ESO dual therapy, the KPZ dual therapy may have a trend of higher *H. pylori* eradication rates in most subgroups.

**Table 2.** Eradication rates of each treatment group.

Analysis	14d-KA group	14d-EA group	Difference from 14d-EA (adjusted 95% CI for difference)	*P* value for difference^a^	*P* value for non-inferiority^b^
ITT	87.9%(116/132)	80.3%(106/132)	7.6% (−1.3% to 16.5%)	0.092	<0.001*
95%Cl	81.2-92.4%	72.7-86.2%			
mITT	92.1%(116/126)	86.2%(106/123)	5.9% (−1.9% to 14.1%)	0.135	<0.001*
95%Cl	86.0-96.0%	79.0-91.2%			
PP	93.5%(116/124)	88.3%(106/120)	5.2% (−2.1% to 13.0%)	0.155	<0.001*
95%Cl	87.8-96.7%	81.3-92.9%			

^a^*p*-values were obtained from two-sided comparisons of the difference between the 14-day KA group and the 14-day EA group.

^b^*p-*values were obtained from one-sided test comparisons of non-inferiority between the 14-day KA group and the 14-day EA group.

*p-value is statistically significant.

### 3.3. Adverse events and compliance

The incidence of adverse events in the KA group and the EA group was 12.1% (16/132) and 16.7% (22/132), respectively, with no statistically significant difference between the groups (*p* = 0.293). Common adverse events included abdominal bloating, diarrhea, nausea/vomiting, etc. (Table 3). The majority of reactions were mild or moderate and gradually disappeared after the end of treatment. In the KA group, 2 patients discontinued medication due to obvious abdominal pain and rash, respectively, but they returned to normal after drug withdrawal. In the EA group, 3 patients discontinued medication due to nausea/vomiting, skin itching, and tinnitus, respectively; among them, the patient who discontinued due to tinnitus had a history of sudden deafness, and all patients returned to normal after drug withdrawal. No serious adverse events occurred in either group during the study. In addition, a total of 5 patients (2 in the KA group and 3 in the EA group) did not take medication regularly due to personal reasons, resulting in a medication proportion of less than 80%. The adherence rates were high in both groups, with the KA group at 97% (128/132) and the EA group at 95.5% (126/132), and there was no statistically significant difference between the groups (*p* = 0.519).

**Table 3.** Adverse events and compliance in the two treatment groups.

	14d-KA group	14d-EA group	
	(*N* = 132)	(*N* = 132)	*p* value
Overall incidence of adverse events	16(12.1%)	22(16.7%)	0.293
Mild	11(8.3%)	14(10.6%)	0.528
Moderate	3(2.3%)	5(3.8%)	0.473
Severe	2(1.5%)	3(2.3%)	0.652
Variety of adverse events			
Abdominal pain	2(1.5%)	3(2.3%)	0.652
Abdominal bloating	3(2.3%)	4(3.0%)	0.702
Diarrhea	2(1.5%)	3(2.3%)	0.652
Constipation	1(0.8%)	2(1.5%)	0.561
Nausea/Vomiting	3(2.3%)	2(1.5%)	0.652
Dry mouth	2(1.5%)	3(2.3%)	0.652
Bitter taste	1(0.8%)	3(2.3%)	0.314
Headache	2(1.5%)	1(0.8%)	0.561
Dizziness	2(1.5%)	1(0.8%)	0.561
Pruritus	0(0.0%)	1(0.8%)	0.316
Rash	1(0.8%)	0(0.0%)	0.316
Tinnitus	0(0.0%)	1(0.8%)	0.316
Dyspepsia	2(1.5%)	4(3.0%)	0.409
Discontinuation due to adverse events	2(1.5%)	3(2.3%)	0.652
Good compliance	128(97.0%)	126(95.5%)	0.519

### 3.4. Risk factors influencing efficacy

Table 4 shows that the factors affecting the eradication efficacy of the two dual regimens were presented in the ITT population. The analysis found that a history of ulcers affected the eradication rate in the EA dual therapy group; while the occurrence of adverse reactions and poor adherence had a negative impact on the eradication rates in both the KA dual therapy group and the EA dual therapy group. In addition, other factors such as gender, age, BMI, BSA, history of alcohol consumption, and digestive system symptoms did not affect the efficacy of either group.

**Table 4.** Univariate analysis of factors influencing eradication rates in the two treatment groups.

Variables	14d-KA group (*N* = 132)		14d-EA group (*N* = 132)	
	Eradication rate	*p*-value	Eradication rate	*p*-value
Gender				
Male	54/63 (85.7%)	0.467	43/56 (76.8%)	0.383
Female	62/69 (89.9%)		63/76 (82.9%)	
Age				
<35	36/43 (83.7%)	0.354	44/50 (88.0%)	0.132
35–50	42/47 (89.4%)		34/45 (75.6%)	
>50	38/42 (90.5%)		28/37 (75.7%)	
BMI, kg/m^2^				
<22	47/54 (87.0%)	0.913	51/63 (81.0%)	0.512
22–25	43/49 (87.8%)		30/39 (76.9%)	
>25	26/29 (89.7%)		25/30 (83.3%)	
BSA, m^2^				
≥1.65	70/81 (86.4%)	0.517	56/72 (77.8%)	0.424
<1.65	46/51 (90.2%)		50/60 (83.3%)	
Marital status				
Married	96/111 (86.5%)	0.466	84/107 (79.0%)	0.405
Unmarried	20/21 (95.2%)		22/25 (88.0%)	
Smoking				
Often	20/25 (80.0%)	0.304	13/16 (81.3%)	1.000
No smoking	85/95 (89.5%)		83/105 (79.1%)	
Drinking				
Often	9/9 (100%)	0.999	6/11 (54.6%)	0.062
No drinking	86/95 (90.5%)		79/98 (80.6%)	
Style of dining				
Gather dining	93/106 (87.7%)	0.999	82/101 (81.2%)	0.644
Individual dining	23/26 (88.5%)		24/31 (77.4%)	
Time of *H. pylori* infection				
> A year	13/17 (76.5%)	0.222	18/23 (78.2%)	1.000
In a month	95/106 (89.6%)		71/90 (78.9%)	
History of ulcers				
Yes	5/8 (62.5%)	0.057	4/8 (50.0%)	0.048*
No	111/124 (89.5%)		102/124 (82.3%)	
History of gastritis				
Yes	34/36 (94.4%)	0.233	26/33 (78.8%)	0.800
No	82/96 (85.4%)		80/99 (80.8%)	
Family history of gastric carcinoma				
Yes	1/2 (50%)	0.229	2/3 (66.7%)	0.485
No	115/130 (88.5%)		104/129 (80.6%)	
History of antibiotics use in 2 years				
Yes	64/75 (85.0%)	0.304	58/73 (79.5%)	0.785
No	52/57 (91.2%)		48/59 (81.4%)	
Digestive symptoms				
Yes	95/108 (88.0%)	0.999	77/97 (79.4%)	0.658
No	21/24 (87.5%)		29/35 (82.9%)	
Side effect				
Yes	10/16 (62.5%)	0.005*	13/22 (59.1%)	0.015*
No	106/116 (91.4%)		93/110 (84.6%)	
Compliance				
Good	116/128 (90.6%)	<0.001*	106/126 (84.1%)	<0.001*
Poor	0/4 (0.0%)		0/6 (0.0%)	

## 4. Discussion

To the best of our knowledge, this is the first multi-center randomized controlled study to compare the efficacy and safety of the KPZ plus amoxicillin dual therapy versus the ESO plus amoxicillin dual therapy for the eradication of *H. pylori* infection. This study confirmed that in terms of efficacy and safety for eradicating *H. pylori*, the KPZ-based regimen was non-inferior to the ESO-based regimen. In the ITT, mITT, and PP populations, the eradication rates of the KA group were 87.9%, 92.1%, and 93.5%, respectively. These results corroborate the sustained acid-suppressive capacity and potential clinical benefit of KPZ demonstrated in preliminary studies. Furthermore, no serious adverse events were reported in either treatment group in this study. We believe that the KPZ-based dual therapy is a viable first-line treatment option for the eradication of *H. pylori*.

The eradication rate of traditional *H. pylori* triple therapy containing PPI continues to decline, presenting a global clinical challenge. The main reasons for eradication failure include antibiotic resistance, poor adherence, low gastric pH, high bacterial load, and rapid metabolism of PPI in the human body. Previous optimizations for *H. pylori* treatment regimens were mostly limited to combinations of antibiotics, probiotics, and bismuth, yet eradication rates still showed a downward trend. With the continuous deepening of understanding regarding the effects of acid suppressants and amoxicillin in recent years, the scientific mechanism of high-dose dual therapy has been further elucidated. According to the 2022 Chinese *H. pylori* guidelines, PPI-based dual therapy has been included as a standard first-line treatment for *H. pylori* infection in China [7]. The clinical efficacy of the ESO-based dual therapy in this study is basically consistent with previous research results [25,26]. Previous studies have reported that the eradication rates of ESO-based dual therapy were 88.5% and 85.5%, respectively. Although approaching 85%, this efficacy still falls short of the ideal 90% target. Previous preclinical studies have confirmed that intragastric pH is closely related to the survival and proliferation of *H. pylori*. Antacids can not only enhance the chemical stability of antibiotics but also improve antibacterial efficacy by regulating gastric pH. Specifically, on one hand, increasing gastric pH can induce *H. pylori* to enter an active proliferation phase, thereby increasing its sensitivity to drugs; on the other hand, a high pH environment can significantly reduce the Minimum Inhibitory Concentration (MIC) of most antibiotics. In addition, antacids have the effect of inhibiting urease activity, thereby weakening the metabolic capacity of *H. pylori* and reducing its colonization in the stomach [27]. In recent years, relevant studies have confirmed that P-CAB acid suppressants are more effective and durable in inhibiting gastric acid secretion than PPIs [28]. P-CAB drugs have a long half-life and do not require repeated administration, whereas PPI drugs have a relatively short half-life and require frequent, high-dose administration, which easily induces or aggravates atrophic gastritis caused by *H. pylori* infection. If P-CAB drugs are used instead of PPIs, it will help reduce this risk [29,30]. In one study, a single 20 mg dose of KPZ inhibited acid secretion more stably and persistently than 30 mg lansoprazole. KPZ maintained intragastric pH > 5 for over 80% of the time (exceeding 95% at night) and had a longer half-life (6.00–7.17h *vs.* 2h), with no pH drop below 6 observed after 16 h. And during medication administration, KPZ is not affected by food intake, providing greater convenience for patients [31]. In contrast, PPIs require food stimulation and cannot completely inhibit nocturnal acid secretion, leading to nocturnal acid breakthrough [32]. Therefore, we believe that in the eradication treatment of *H. pylori*, KPZ can replace PPI to solve the current problem of declining eradication rates.

Amoxicillin, as a pH-dependent antibiotic, shows stronger bactericidal efficacy at higher gastric pH, and being time-dependent, its efficacy is enhanced by high-dose, high-frequency administration [16,33]. In addition, apart from allergies, amoxicillin has the fewest side effects and almost no impact on the gut microbiota [34]. Given that KPZ possesses potent and sustained acid-suppressive properties, capable of creating an ideal environment for amoxicillin activity to maximize antibacterial effects. We think that KPZ combined with high-dose amoxicillin dual therapy has significant potential advantages for eradicating *H. pylori* without the need for susceptibility testing.

Antibiotic resistance is a key factor affecting the efficacy of first-line treatment [35,36]. This study explored whether antibiotic use within the two years prior to treatment affected the eradication efficacy in the KPZ dual therapy group and the ESO dual therapy group. The results showed that although the eradication efficacy of the KPZ dual therapy group was not statistically different, it was numerically superior to that of the ESO dual therapy group. This suggests previous antibiotic exposure induces resistance, reducing efficacy, while KPZ dual therapy may increase *H. pylori* susceptibility to improve eradication rates. This finding is consistent with previous conclusions that therapies containing P-CAB drugs can overcome antibiotic resistance and thereby improve eradication rates [37,38]. Given that antibiotic susceptibility testing is invasive, costly, and complex, most international consensus guidelines do not recommend routine testing before treatment. Encouragingly, amoxicillin’s low resistance rate in the Asia-Pacific region eliminates the need for pre-treatment susceptibility testing, reducing the medical burden [39,40]. However, regardless of the regimen, clinicians should inquire about patients’ antibiotic history to avoid reusing frequently exposed antibiotics for *H. pylori* treatment.

Notably, our subgroup analysis revealed a significant difference in eradication rates between the KA and EA groups among alcohol drinkers. This suggests that alcohol may exacerbate the inherent limitations of PPI previously discussed. Since the activation and metabolism of PPIs depend on CYP2C19, alcohol-induced acid hypersecretion and accelerated CYP2C19 metabolism can easily offset the acid-suppressive effect of PPIs, thereby diminishing amoxicillin efficacy [41–45]. In contrast, KPZ is unaffected by CYP2C19; its potent, sustained acid suppression may directly counteract alcohol-stimulated acid secretion, thereby maintaining gastric pH stability and ensuring amoxicillin efficacy. This finding is consistent with previous research indicating that alcohol does not adversely affect treatment regimens based on VPZ, another P-CAB [46]. However, due to the relatively small sample size of this subgroup, this finding should be interpreted with caution, and future studies should further focus on exploring the underlying mechanisms by which KPZ-based therapies remain unaffected by alcohol consumption.

Additionally, a history of ulceration affects the eradication rate in the EA group. This aligns with previous studies reporting a significantly lower eradication rate in the scarring stage of gastric ulcers [47]. Healed ulcers permanently retain fibrotic scars and glandular atrophy, which not only disrupt normal acid regulation but may also physically impede the local diffusion of antibiotics. We speculate that in such compromised mucosa, the unstable acid suppression by PPI may be insufficient to overcome this dysregulation, leading to pH fluctuations that attenuate amoxicillin efficacy. Conversely, the potent acid suppression of KPZ may bypass this mucosal defect to maintain stable pH levels. However, given the borderline p-value, this finding should be interpreted with caution and awaits further validation by future studies.

The duration of therapy is a critical factor for the successful eradication of *H. pylori*. Currently, most guidelines and the HDDT recommend a standard 14-day course. Previous clinical studies have shown that the eradication rates of 10-day PPI dual therapy are often unsatisfactory [48]. However, a multi-center randomized clinical study conducted in China compared the clinical efficacy and safety of 10-day VA dual therapy with rabeprazole combined with amoxicillin dual therapy (RA) [13]. The results showed that the *H. pylori* eradication rate in the VA group was significantly better than that in the RA group (91.4% vs. 86.6%, *p* < 0.05). Despite this, at the initial design of this study, clinical studies on the 10-day short course of P-CAB drugs were relatively few, and clinical evidence was insufficient. Furthermore, considering the relatively high antibiotic resistance rate in the Gansu region of China, in order to balance clinical research exploration with ensuring patient eradication rates, we adopted a relatively conservative strategy and selected the 14-day course recommended by current guidelines. It is gratifying that the 14-day KPZ dual therapy achieved satisfactory eradication rates without serious adverse events. Furthermore, Previous data indicate that KPZ demonstrates superior acid-suppressive effects compared to VPZ. Specifically, KPZ achieved significantly higher HTR% (pH >4) on both day 1 and day 7, and maintained 24-hour and nighttime HTRs consistently above 99% with a more stable acid suppression curve [49–51]. Thus, we hypothesize that the 10-day KPZ dual therapy may offer comparable eradication efficacy to VPZ, pending further clinical verification.

This study did not identify any serious adverse events related to KPZ. The adverse reactions that occurred were relatively consistent with results observed in previous studies on KPZ [22,52]. Our study confirmed the safety of the KPZ-based dual therapy regimen in the treatment of *H. pylori* eradication. Furthermore, our analysis indicated a positive correlation between the occurrence of adverse reactions and eradication failure (*p* < 0.05). Although the drug composition is relatively simple, adverse reactions remain a potential risk, interfering with treatment continuity. During our actual follow-up process, we observed that the complexity of this issue in clinical reality far exceeds the data itself. Specifically, anxiety over mild-to-moderate adverse reactions often leads to premature self-discontinuation and treatment failure. This highlights the need for proactive patient education, as informing patients that side effects are typically transient and providing clear management strategies can minimize the impact of adverse reactions on eradication rates.

Poor patient adherence is a significant cause of eradication failure, a conclusion consistent with the results of multiple previous studies [13,53]. A high pill burden, complex administration regimens, and obvious drug side effects are the main factors that weaken adherence. Notably, although the KPZ-based dual therapy regimen in our study demonstrated excellent overall adherence, reaching 97% and indicating good tolerability and clinical potential, successful eradication was still not achieved in some individuals with poor adherence. We speculate that the duration of therapy may have become a new sensitive factor affecting patients’ continued medication use. In summary, we once again propose a hypothesis: for the 14-day KPZ dual regimen, is it possible to optimize the duration by reducing it to 10 days? However, it requires the consideration of more factors. For example, further research is needed on specific populations (the elderly, immunocompromised patients, and patients with refractory *H. pylori* infection).

Dual therapy combining P-CABs and amoxicillin shows variable efficacy depending on the specific P-CAB used. The VPZ-amoxicillin regimen is well-established with an eradication rate of over 90% [54,55]. Our study also showed an eradication rate of 93.5% for the KPZ regimen, which is consistent with previous findings on the KPZ dual therapy [56]. In contrast, the TPZ regimen yielded lower rates (72.59% & 81.0%) [57,58]. We attribute this discrepancy to pharmacokinetic and pharmacodynamic differences: KPZ and VPZ have longer half-lives and stronger acid suppression than TPZ [31,50,59]. TPZ’s lower efficacy may also stem from its benzimidazole structure, different H^+^/K^+^-ATPase binding sites, as well as variations in antibiotic resistance rates and the MIC distribution of *H. pylori* [60]. Future head-to-head RCTs are needed to directly compare these P-CABs and optimize TPZ dosing.

P-CAB-based dual therapy shows regional variability, yielding suboptimal eradication rates in Western countries and South Korea [38,61]. In Western populations, despite low amoxicillin resistance, the high prevalence of obesity increases the volume of antibiotic distribution, leading to subtherapeutic drug concentrations [62–67]. Additionally, differing administration timings (pre-meal *vs.* post-meal) may affect gastric antibiotic exposure [28]. In South Korea, the suboptimal efficacy may be primarily attributed to two factors. First, its historical amoxicillin resistance breakpoint is more lenient (≥0.5 µg/mL) than the strict EUCAST standard (>0.125 µg/mL), which may mask a higher actual resistance rate and undermine dual therapy efficacy [68,69]. Second, South Korean studies predominantly use TPZ, which has a shorter half-life. The conventional 50 mg bid dosage struggles to maintain 24h acid suppression, leading to nocturnal acid breakthrough that limits the efficacy of amoxicillin, an antibiotic highly dependent on elevated intragastric pH [70]. In conclusion, future research in Western countries and South Korea must further investigate the optimal dosing and duration of various P-CAB-based dual therapies, and thoroughly evaluate their efficacy and safety in specific populations.

In the eradication treatment of *H. pylori*, the application of antibiotics is indispensable, but their negative impact on the body’s microecology has become a clinical issue that cannot be ignored. Existing evidence indicates that while antibiotics eliminate pathogenic bacteria, they also widely disrupt the homeostasis of the gut microbiota and weaken the colonization resistance of normal commensal bacteria [71]. A deeper concern lies in the long-term alterations in gut microbiota structure after *H. pylori* eradication, which may be potentially associated with the pathogenesis of pseudomembranous colitis and inflammatory bowel disease [72,73]. Previous studies have compared the effects of different eradication regimens on the gut microbiota. The results showed that compared with traditional triple therapy and bismuth quadruple therapy, dual therapy causes relatively less damage to the diversity and structure of the gut microbiota due to the use of one fewer antibiotic, demonstrating better potential for microecological protection [74,75]. Among the components of eradication regimens, in addition to the types and quantities of antibiotics, the type, dosage, and duration of use of acid suppressants are also considered key variables affecting the gut microecology [76].

However, differences in the pharmacological characteristics of the acid suppressants themselves may also lead to distinct microecological outcomes. Taking P-CAB drugs as an example, although this class possesses the advantages of rapid onset and potent acid suppression, their impact on the gut microecology remains controversial. Currently, relatively numerous studies have been conducted on VPZ, with many studies demonstrating that VPZ causes greater disruption to the gut microbiota. For instance, in both healthy populations and patients treated with aspirin, VPZ has shown more significant gut microbiota dysbiosis than PPIs [77,78]. Another study has pointed out that VPZ may aggravate small intestinal injury by reducing the quantity of Lactobacillus johnsonii in the small intestine [79]. But a study by Son et al. provided another perspective. They found that TPZ, also a P-CAB, was able to enhance intestinal barrier function, promote beneficial bacteria growth, and inhibit pathogenic bacteria in a mouse model, thereby reducing inflammation and effectively preventing the occurrence of colitis [80]. This finding suggests that different P-CAB molecular structures may exert distinct or even opposite regulatory effects on the gut microbiota. In summary, dual therapy is preferable for eradicating *H. pylori* to minimize its impact on the gut microbiota. Since the effect of KPZ on gut microbiology has not been studied, further exploration is needed.

This study has certain limitations. Firstly, this study only collected the history of previous antibiotic use for each patient at baseline, and did not conduct antibiotic susceptibility testing or genotypic resistance testing. Therefore, we cannot accurately assess the impact of amoxicillin resistance on eradication efficacy. Future research involving further studies, such as susceptibility testing, would better explain the clinical results. Secondly, individuals over the age of 65 were excluded. Whether elderly patients should be treated remains controversial, and their optimal treatment regimen requires further investigation. Thirdly, this study utilized only amoxicillin as the antibiotic, limiting its application in the penicillin-allergic population. Previous studies have shown that VPZ combined with tetracycline achieved satisfactory eradication efficacy (≥90%) with good safety in penicillin-allergic treatment patients [81,82]. Future research could further explore combination regimens of KPZ with other antibiotics, evaluate their eradication effects in different *H. pylori*-infected population, and investigate the impact of these novel treatment regimens on the gut microbiota. Finally, due to the non-inferiority design of the study, future clinical studies with larger sample sizes are needed to confirm the superiority of the KPZ dual regimen over the ESO dual regimen in the eradication of *H. pylori*.

## 5. Conclusion

For treatment-naive patients with *H. pylori* infection, the 14-day KA dual therapy is non-inferior to the 14-day EA dual therapy, and the KPZ dual therapy demonstrates good tolerability and a favorable safety profile. Given its pharmacogenomic independence and potent acid suppression, the KA dual therapy can be considered a rational first-line alternative to EA therapy in the Chinese population.

## Supplementary Material

CONSORT 2025 checklist.docx

## Clinical trial registration

This trial has been registered at ClinicalTrials.gov (Registration No. NCT06851468).
